# Parental factors that impact the ecology of human mammary development, milk secretion, and milk composition—a report from “Breastmilk Ecology: Genesis of Infant Nutrition (BEGIN)” Working Group 1

**DOI:** 10.1016/j.ajcnut.2022.11.026

**Published:** 2023-05-10

**Authors:** Margaret C. Neville, Ellen W. Demerath, Jennifer Hahn-Holbrook, Russell C. Hovey, Jayne Martin-Carli, Mark A. McGuire, Edward R. Newton, Kathleen M. Rasmussen, Michael C. Rudolph, Daniel J. Raiten

**Affiliations:** 1 Department of Obstetrics and Gynecology, University of Colorado, Aurora, CO, USA; 2 Division of Epidemiology and Community Health, University of Minnesota, Minneapolis, MN, United States; 3 Department of Psychological Sciences, University of California Merced, Merced, CA, United States; 4 Department of Animal Science, University of California Davis, Davis, CA, United States; 5 Department of Pediatrics, University of Colorado, Aurora, CO, United States; 6 Idaho Agricultural Experiment Station, University of Idaho, Moscow, ID, United States; 7 Department of Obstetrics and Gynecology, Brody School of Medicine, East Carolina University, Greenville, NC, United States; 8 Nancy Schlegel Meinig Professor of Maternal and Child Nutrition, Division of Nutritional Sciences, Cornell University, Ithaca, NY, United States; 9 The University of Oklahoma Health Science Center, Oklahoma City, OK, United States; 10 Pediatric Growth and Nutrition Branch, *Eunice Kennedy Shriver* National Institute of Child Health and Human Development, National Institutes of Health, Bethesda, MD, United States

**Keywords:** human milk, milk secretion, chronobiology, oxytocin, obesity, diabetes, membrane transporters, model systems, microbiome, environmental toxins

## Abstract

The goal of Working Group 1 in the Breastmilk Ecology: Genesis of Infant Nutrition (BEGIN) Project was to outline factors influencing biological processes governing human milk secretion and to evaluate our current knowledge of these processes. Many factors regulate mammary gland development in utero, during puberty, in pregnancy, through secretory activation, and at weaning. These factors include breast anatomy, breast vasculature, diet, and the lactating parent’s hormonal milieu including estrogen, progesterone, placental lactogen, cortisol, prolactin, and growth hormone. We examine the effects of time of day and postpartum interval on milk secretion, along with the role and mechanisms of lactating parent-infant interactions on milk secretion and bonding, with particular attention to the actions of oxytocin on the mammary gland and the pleasure systems in the brain. We then consider the potential effects of clinical conditions including infection, pre-eclampsia, preterm birth, cardiovascular health, inflammatory states, mastitis, and particularly, gestational diabetes and obesity. Although we know a great deal about the transporter systems by which zinc and calcium pass from the blood stream into milk, the interactions and cellular localization of transporters that carry substrates such as glucose, amino acids, copper, and the many other trace metals present in human milk across plasma and intracellular membranes require more research. We pose the question of how cultured mammary alveolar cells and animal models can help answer lingering questions about the mechanisms and regulation of human milk secretion. We raise questions about the role of the lactating parent and the infant microbiome and the immune system during breast development, secretion of immune molecules into milk, and protection of the breast from pathogens. Finally, we consider the effect of medications, recreational and illicit drugs, pesticides, and endocrine-disrupting chemicals on milk secretion and composition, emphasizing that this area needs much more research attention.

## Introduction

The “Breastmilk Ecology: Genesis of Infant Nutrition (BEGIN)” Project was designed to: *1*) examine the ecology of human milk, based on the supposition that human milk represents a complex biological system that interacts with both the internal biology and health of the lactating person, the human milk matrix, and the impact on the breastfed infant and external (social, behavioral, cultural, and physical) environments (see Text Box 1 for Core Concepts and Terms); *2*) explore the functional implications of this ecology for both the biological parent and their infant; and *3*) explore ways in which this emerging knowledge can be studied and expanded via a targeted research agenda and translated to support the community’s efforts to ensure safe, efficacious, equitable, and context-specific infant feeding practices in the United States and globally.

Text Box 1 **Core Concepts and Terms.**

• In the context of this paper, “ecology” is defined as a complex biological system and its interactions with its environment. In this case, the complex system is human milk composition and its inherent biology, and the environment consists of parental and infant inputs and the influence of their respective internal and external environments.
• With due recognition of the need to be observant of issues of gender identity/neutrality, and to improve precision, to the extent possible, for the purposes of the papers described herein, we will use gender neutral terminology where appropriate (e.g., lactating parent/person etc.), to reflect the reality that not all who lactate identify as female. The term “lactating parent” respects and recognizes those who may have been born female but do not identify as such as well as other gender-relevant contingencies. In situations where reporting primary data (studies/analyses), we will refer to the population as specified (e.g., “the study evaluated 250 lactating mothers”). Moreover, rather than using terms such as “maternal” or “maternal milk,” we will use terms such as “birthing parent” throughout the report as appropriate as they accurately reflect the biological nature of the birthing parent-infant dyad.
• “Human milk” refers to milk produced by lactating parents and includes both: *1*) breastmilk produced by a parent for their infant and fed directly to infants via the breast or expressed by the lactating parent and then fed to the infant; and *2*) donor/banked human milk produced by lactating persons that is either donated to human milk banks or fed to infants other than their own child.

Alt-text: Text Box 1

The overarching conceptual framework and description of the Project is presented in the BEGIN executive summary, the first of 6 manuscripts of this supplement [1]. Our hope is that the reader will also review the subsequent manuscripts in this supplement, which present the findings of the individual thematic BEGIN working groups (WGs) as a continuum of thought that reflects a larger conceptual view of how we can move this important research and public health agenda forward.

Specifically, the BEGIN Project was accomplished by forming five thematic WGs charged with addressing the following themes: *1*) parental inputs to human milk production and composition; *2*) the components of human milk and the interactions of those components within this complex biological system; *3*) infant inputs to the matrix, emphasizing the bidirectional relationships associated with the breastfeeding dyad; *4*) the application of existing and new technologies and methodologies to study human milk as a complex biological system; and *5*) approaches to translation and implementation of new knowledge to support safe and efficacious infant feeding practices. This article represents the results of the deliberations of WG 1.

The main task assigned to WG 1 was to address the key question: “What are the parental factors that affect the composition and volume of breastmilk?” As the group began to address this question it became clear that additional questions needed to be addressed:

• How does mammary gland development, particularly during pregnancy, affect the ability of the gland to secrete milk?
• What is the input of the infant to the process of secretory activation and milk secretion?
• How do disease conditions, inflammation, preterm birth, and particularly, obesity, impact the process?
• How do environmental agents, medications, and recreational drugs affect the processes of mammary development and milk secretion?

**FIGURE 1:**
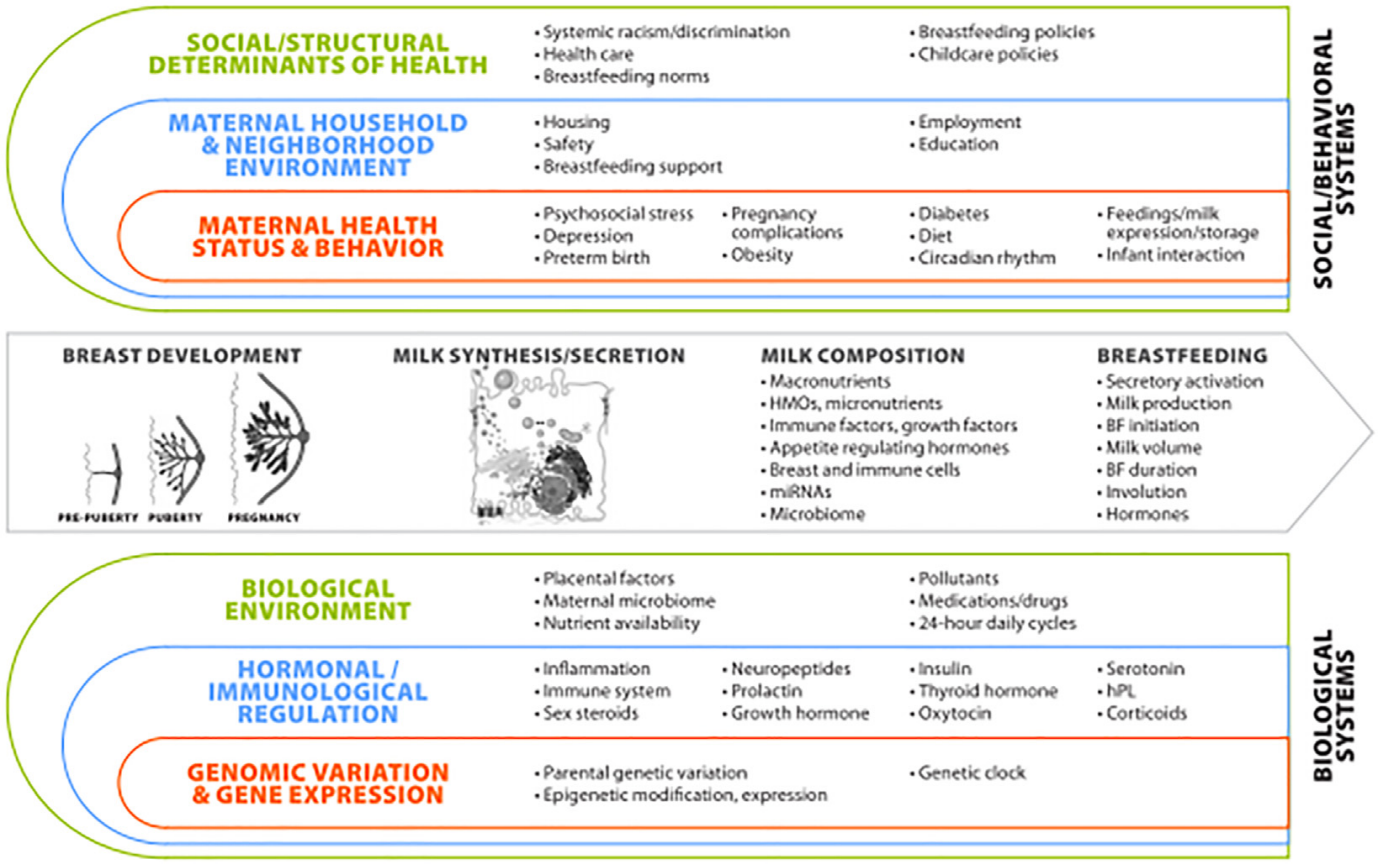
The breast system: inputs and outputs. BF, breastfeeding; hPL, human placental lactogen; HMO, human milk oligosaccharide.

We share a pictorial overview of the WG 1 scope in Figure 1, which illustrates the plethora of biological and social/behavioral factors that may impact breast development, milk secretion, and milk composition. We begin by addressing the factors that regulate normal mammary gland development, then consider many of the inadequately understood issues that impact milk volume and composition in the healthy individual, including breast anatomy, the vascular system, the hormonal milieu, and diet as well as time of day and postpartum interval. After considering the role and mechanisms of parental-infant interactions in milk secretion and bonding, we consider the impact of parental health on breast development, including the effects of infection, preterm birth, cardiovascular health, inflammatory states, and particularly, gestational diabetes and obesity. Throughout all phases of mammary development and lactation the immune system is lurking, potentially impacting breast development, directing the secretion of immunoglobulins into milk, protecting the breast from foreign agents, and doing things we have yet to imagine. Finally, an area that has received little attention in recent years for its impact on breastfeeding success is the effect of environmental agents including medications, recreational and illicit drugs, pesticides, and endocrine-disrupting chemicals (EDCs).

## Breast Development

Text Box 2 shows the important developmental stages of the breast that are summarized in this section of the report.

Text Box 2 **Developmental stages in the human breast.**

1. Embryonic development.
2. Ductal development during puberty.
3. Development of the acinar epithelium during pregnancy and secretory differentiation.
4. Secretory activation after parturition.
5. Involution after weaning.

Alt-text: Text Box 2

### The mammary gland in the newborn

At birth, the normal human breast is represented by a 1-cm bud [2] consisting of a nipple and a rudimentary mammary tree in the interstitial space [3]. In some infants a small amount of milk may be secreted postpartum, after which isometric growth of the quiescent gland occurs until puberty [4].

### Ductal development at puberty

At puberty, increased circulating estrogens from the ovaries are the major stimulator of ductal development, acting on epithelial estrogen receptor alpha (ERα). IGF1, secreted by the liver or locally by stromal cells under the direction of growth hormone (GH), also fosters branching morphogenesis of the ductal system and the formation of terminal end buds that direct the ducts through the growing fat pad [3]. A host of growth factors such as amphiregulin [5] or adipose-derived agents such as leptin [6] also likely play a role in ductal development during puberty. Once menarche occurs, progesterone secretion during the luteal phase of the ovarian cycle further stimulates formation of terminal duct lobular units (TDLUs). This phase of breast development often occurs when the adolescent is encountering a range of lifestyle factors including obesity, recreational drug use, or excess alcohol intake, all against the backdrop of a consistently declining age of puberty [7].

### Development of the alveolar epithelium in pregnancy

The ductal epithelium has a basal layer that will become the myoepithelium responsible for milk ejection and a luminal layer whose cells will secrete milk in the mature functional gland. In the first trimester of pregnancy, human chorionic gonadotropin (hCG) acts on the corpus luteum in the ovary to maintain and increase the secretion of estrogen and progesterone, both necessary for placental and mammary gland development [8,9]. After the first trimester the placenta becomes the major driver of mammary gland growth and differentiation, when the syncytiotrophoblasts lining the maternal blood vessels in the placenta take over the secretion of estrogen and progesterone. These cells also secrete human placental lactogen (hPL), a member of the prolactin (PRL)-GH family. During pregnancy, hPL can be found in the parental urine at concentrations ∼25 times that of PRL [10]. hPL is thought to bind the PRL receptor (PRLR), although its effects on alveolar differentiation are not fully understood. Likewise, a well established yet overlooked consideration is that hGH is clearly lactogenic through its actions on the PRLR [11]; hGH is preferentially secreted by the placenta as hGH-V placental GH [12].

Estrogen and progesterone are the major drivers of the massive increase in epithelial development during pregnancy, where they interact with ERα and both progesterone receptors A and B (PRA and PRB), respectively [[13], [14], [15]]. Progesterone may act in a paracrine fashion causing PR-positive cells to secrete growth factors that stimulate proliferation in neighboring cells, although more work is needed to verify this mechanism in humans [13]. At the same time, progesterone also inhibits milk secretion, allowing the gland to accumulate colostrum, which resides in the ductal and alveolar lumen until birth, potentially protecting the gland against infection.

The placenta is also involved, secreting exosomes, IGF binding protein 2 (IGFBP2), and many cytokines and growth factors. It is becoming clear that conditions like obesity and gestational diabetes alter the secretion of these components [16,17], thereby affecting the metabolism of diverse and distant organs. The effect of these conditions on lactation will be addressed in section IV, and additional questions about EDC will be addressed in section VI. Important emerging questions are provided in Text Box 3, as modified from Lee and Kelleher [18].

Text Box 3 **How do clinical conditions modify mammary development in human pregnancy? While these questions have been addressed in animals, they have not been adequately addressed in humans.**

• Do genetic variations in major milk proteins or genes for regulatory proteins like IGF1 or FGFs modify the processes of mammary development or milk production?
• What is the role of diet in maintaining maternal energy balance and the hormones of pregnancy?
• What are the effects of obesity on mammary development and secretory activation? Does it produce defects in alveolar development, the extracellular matrix, or the myoepithelium?
• Are there effects of an inflammatory environment on the developing breast and its future function?
• Could severe caloric restriction during pregnancy release fat-soluble environmental contaminants and toxins, including polychlorinated biphenyls (PCBs) and pesticides that are stored in body fat, to have effects on mammary development?
• Could preeclampsia or energy/nutrient imbalance affect the immune system to increase the risk of mastitis or otherwise alter the effects of immune cells on mammary development and lactation success?
• Are there additional placental or decidual factors such as IGFBP1, placental exosomes, adiponectin, or other placental cytokines that alter development of the mammary secretory apparatus?

Alt-text: Text Box 3

## Factors That Influence the Normal Secretion of Milk: Anatomy, Hormones, Homeorhesis, and Chronobiology

### Breast anatomy

The developing breast has 4 to 18 lobes (average 9) composed of a ductal network that drains acini composed of two layers of cells: milk secreting cells that surround a lumen into which they secrete milk from their apical surfaces and peripheral myoepithelial cells that contract during the letdown reflex. Ejected milk then drains into the lactiferous ducts that transport it to the nipple [19]. These acini vary in their morphology, where the predominant subtype in the lactating breast has a large milk-filled lumen with flattened epithelial cells [20]. The secretory lobules are also embedded within a complex stromal tissue composed of inter- and intralobular connective tissue [21]. White adipocytes, whose extent is highly variable among women, have a remarkable phenotypic plasticity and are thought to play a role in the development and function of the mammary epithelium [22,23]. About 4% of women have severe hypoplasia or insufficient glandular tissue, often manifesting as tubular breasts; other anatomical pathologies severe enough to impact breastfeeding success include ptosis, inverted nipples, and breast asymmetry [24]. As a parallel to breastfeeding biology, mammographic screening for breast cancer provides a measure of radiographic density, a proxy for examining epithelial/stromal tissue. This technique might also offer insight into the effects of breast stroma on mammary development. Combined, there is the need to better understand normal breast variation, from the genetic through the endocrine to the histologic front. Animal models such as pigs and non-human primates may be useful in this regard [25,26].

### Secretory systems in the mammary epithelial cell

Five classical pathways are used by mammary epithelial cells (MECs) for the secretion of milk components [27]. Pathway 1 is the Golgi-secretory vesicle pathway responsible for lactose, casein, α-lactalbumin, lactoferrin, oligosaccharides, and other constituents of the aqueous fraction of milk. Pathway 2 involves the milk fat globules (MFGs) that contain the lipid portion of milk, as well as proteins such as butyrophilin (BUT1A1), mucins 1 and 4, xanthine oxidoreductase, adipophilin (perilipin 2), and lactadherin (MFG-E8) [28,29]. Interestingly, the MFG often contains a fragment of cytoplasm that can be used to analyze the RNA composition of the milk secreting cell [30,31]. Pathway 3 ferries mono- and divalent ions as well as substrates like glucose and amino acids across both basal and apical membranes [32]. Pathway 4 is a direct vesicular pathway mainly involved in transferring IgA from the interstitial space where it is manufactured by resident B cells across the cytoplasm to be secreted as secretory IgA into the milk [33]. The fifth pathway, as yet not as well defined, secretes small extracellular vesicles of various sizes including exosomes into milk [34,35]. Although there is evidence that these vesicles carry signals that alter infant biology, very little is known about their composition, secretion, and effects in the infant [36].

A sixth pathway, the paracellular pathway, operates only during pregnancy, inflammation, and potentially involution, when interstitial molecules like sodium and chloride ions, serum albumin, and luminal molecules like lactose move down a concentration gradient between the alveolar cells during the colostral phase. Closure of the tight junctions that control this paracellular pathway is responsible for most of the decrease in sodium and chloride and the increase in lactose in the early stages of secretory activation.

Transfer of monovalent and divalent ions into milk involves transporters in many of the compartments of the MEC. Traditional studies of ion transport involved following isotope labeled ions and substrates as they entered, transited, and exited MEC. Unfortunately, transporters identified in this way had a variety of often-confusing nomenclatures. Given that the mRNA profile of the lactating human gland has recently become available from analysis of the milk fat globule transcriptome [29,30], transcriptomic techniques have been used to determine which transporters are present for almost any substrate. In addition, identified transporters can be localized within the cell using immunohistochemistry. From these and other techniques we know a great deal about calcium [37,38] and zinc [39,40] transport from the plasma to the milk. In general, the molecules and pathways that transfer copper, amino acids, and glucose into the MEC and across intracellular membranes are well-characterized, and the identity of the numerous channels, co-transporters, and exchangers is known. How these transporters work and interact as well as their localization within the cell require additional work.

### Endocrine regulation of milk secretion

It is clear from experiments in mice and in vitro systems that PRL can regulate all aspects of milk synthesis and secretion [15,41]. PRL may also have metabolic effects on diverse organs throughout the body and likely contributes to the infertility of lactation [42]. In human plasma, PRL is high at parturition, and its action, along with a fall in progesterone, is thought to bring about secretory activation. The PRL inhibitor bromocriptine decreases milk secretion; however, it can have severe side effects and is no longer approved for this purpose [43]. However, the importance of PRL in maintaining human lactation is called into question by a number of observations. In several early studies [[44], [45], [46]], milk volume in exclusively breastfeeding parent-infant pairs remained high (670–896 ml/h) for the first 6 mo of breastfeeding, despite PRL concentrations falling from ∼120 ng/mL at 1 mo to 59 ng/mL at 6 mo. Drugs like domperidone and metoclopramide that augment PRL secretion have been used to increase milk supply in humans [47], but the increase in milk volume is small and their use is no longer recommended in the United States [48]. Finally, the regulation of PRL secretion is highly complex. It is subject to active inhibition by dopamine in non-lactating individuals, but the mechanisms by which its secretion is increased and its importance in human lactation are still unclear [42].

Many other hormones play a role during established lactation including insulin, cortisol, thyroid hormone, and possibly serotonin and hGH (Figure 1). Cortisol has a clear circadian rhythm that may translate into diurnal changes in milk composition (see below). However, exogenous glucocorticoids also have a pronounced suppressive effect on milk secretion [49], possibly underlying stress-induced suppression of lactation. Oxytocin, stimulated by the suckling infant, is important for milk letdown and, as described later, interacts with pleasure centers in the brain to augment positive interactions between the lactating parent and infant [50].

### Effects of breastfeeding on cardiac function

The breasts of an exclusively breastfeeding parent are a metabolic powerhouse, utilizing as much as 20% to 30% of the body’s daily energy production and cardiac output [[51], [52], [53]]. Interrogation of the major arteries supplying the lactating breast by Doppler ultrasound may prove to be a powerful tool in examining changes in cardiovascular hemodynamics during lactation [52]. There is a need for carefully collected measures of basic function including heart rate, blood pressure, urinary output, and respiration rate during lactation for inter- and intrafeeding/pumping episodes and across the epochs of lactation, initiation, transition, full exclusive breastfeeding, introduction of complementary foods, and weaning.

### Homeorhesis

Homeorhesis is the coordinated control of nutrient metabolism in the breastfeeding parent and includes the adaptation of many organs to support the availability of nutrients for milk synthesis [54]. To evaluate homeorhesis in humans it is necessary to know the mass uptake of milk constituents into and transfer out of the gland in addition to concentrations in plasma, mammary cells, and milk; as a priority, the volume of milk transfer to the infant must also be measured, a much more difficult undertaking in lactating parents as outlined in the report of WG 3 [55]. To summarize here, the only two accurate methods of measuring milk transfer to the breastfeeding infant are test-weighing before and after each feed for ≥24 h [56] and deuterium dilution [57,58]. It is important to note that deuterium dilution only gives accurate values if milk volumes are stable, ruling out its use in studying secretory activation and short-term interventions to increase milk volume.

#### Diet

Whereas the total protein and lactose content of human breastmilk varies substantially across different human populations, the level of nutrition, acute dietary changes, and protein and energy supplementation of undernourished individuals has only a limited impact on the concentrations of protein or lactose in breastmilk. By contrast, the concentration of total milk lipids and individual FAs in breastmilk is diet dependent [59]. Dietary FAs, particularly medium- and longchain FAs, are readily incorporated into milk fat, changing their profile in human milk [60,61]. There is no evidence to support the dietary regulation of cholesterol concentration in milk, although changes in total lipid concentration likely reflect changes in all lipid classes, as phospholipids, sphingolipids, and cholesterol are all secreted as part of the MFG.

#### Carbohydrate synthesis

Carbohydrates constitute around 7% of breastmilk and are critical not only for their nutritive value to the infant but also because human milk oligosaccharides (HMOs) are increasingly recognized as important in establishing both the barrier properties and microbiome of the infant gastrointestinal epithelium [62,63] (See report of WG 2 [64]). It has long been known that the osmotic properties of lactose determine milk volume [65,66]. More recently, fructose has been detected in human milk due to the substantial intake of high fructose corn syrup in western societies, even though it is normally absent from breastmilk [67]. The utilization of non-glucose substrates such as glycerol for lactose synthesis also needs more consideration [68]. That said, the synthesis of lactose from hexose substrates, derived either from the diet or through gluconeogenesis, is remarkably stable across different metabolic states, highlighting the insulin-independent nature of lactose synthesis by the mammary gland [69]. One key driver of lower milk production due to impaired lactose synthesis is likely to be stress, given that *α*-lactalbumin gene expression is suppressed by corticosteroids in mice [49,70]. Further, the genetic, as opposed to dietary, regulation of HMO synthesis is well established; recent evidence [71] suggests the potential for dietary regulation of specific HMO groups (i.e., HMO-bound fucose or sialic acid).

#### Other nutrients

The transfer of all vitamins into milk from the blood stream is influenced by their form and their binding proteins. In addition, body stores of fat-soluble vitamins impact the concentration available for incorporation into milk fat, making it difficult to examine the response to acute dietary changes. When individuals with chronic low consumption of vitamin A, D, or E take appropriate supplements, the respective concentration in milk can be increased [72]. Given a lack of body reserves, increases in water-soluble vitamins can be more easily discerned after supplementation [73]. In general, the concentrations of minerals such as calcium, magnesium, sodium, potassium, iron, chlorine, iodine, fluorine, zinc, copper, and manganese in human milk have not been shown to be affected by their dietary intake nor has that of phosphorus. Selenium intake has been shown to impact its concentration in milk; in addition, the form consumed (i.e., selenomethionine compared with selenite) does affect selenium incorporation into milk [74].

#### The intestinal microbiome

Considerable attention has also been directed toward how the intestinal microbiome can affect milk composition through the gut-mammary axis [[75], [76], [77], [78]]. Human milk has its own microbial composition that has been related to BMI as well as macronutrient and micronutrient intake [79]. The presence of various bacterial taxa also reflects dietary intake of FAs, carbohydrates, and protein [80]. Further, the consumption of probiotic foods can lead to the transfer of bacteria into the human milk microbiota [81]. The interaction of HMOs with the infant microbiome is considered in the report of WG 2 [64], and the role of the infant microbiome in infant development is considered in the report of WG 3 [55].

### Chronobiology

Most biological processes in the human body follow a 24-h circadian rhythm, and the mammary gland is no exception. Circadian biology influences breast development [82] as well as the secretion of milk [83,84]; 7% of genes in lactating MEC have a circadian pattern of expression [83,31]. Moreover, knocking out the central circadian clock in mice disrupts mammary development and function [85]. In line with these findings, breastmilk changes dramatically over the day in terms of its nutritional, hormonal, and immunologic composition [86]. As examples, the concentrations of tryptophan, fats, triacylglycerol, cholesterol, iron, melatonin, cortisone, and cortisol in human milk all have circadian patterns [87]. Human milk also contains higher nocturnal concentrations of 5′AMP and 5′GMP—purine nucleotides known to be important for the release of GABA and melatonin, respectively [88]. Activity-promoting neuroactive amino acids (e.g., tyrosine, methionine, phenylalanine, aspartic acid, and glycine) are all at peak concentrations in milk synthesized during the daytime compared to that synthesized at night [89]. Circadian changes in milk composition can also reflect circadian changes in circulating hormones like cortisol and melatonin, which possibly alter receptor-mediated transport of substrates by MEC [90]. Such regulation may explain why daily changes in milk composition (for example, iron concentrations) do not always track with changes in maternal plasma [91]. These diurnal changes in human milk composition are likely regulated, in part, by the suprachiasmatic nucleus, the central clock in the brain [92]. The primary regulator of the central clock is light, although other factors can phase-shift the central clock, for example, staying up all night, altered meal times, medications, or health problems like obesity or stress [93,94]. These factors might also influence the mammary circadian clock and, hence, milk composition. Such a finding would align with findings from work in animals showing that the mammary circadian clock impacts milk yield and composition [83]. A large number of questions remain about which factors influence the normal secretion of milk; see Text Box 4.

Text Box 4 **Factors that influence the normal secretion of milk.**

• **Anatomy**. Does radiographic density afford a unique opportunity to relate the composition of the stromal matrix to breastfeeding outcomes as it does with breast cancer initiation?
• **Anatomy**. Several candidate single nucleotide polymorphisms (SNPs) associate with measures of breast density. Many of these SNPs are in genes known to regulate mammary development and differentiation. Do such observations open the door to decipher the undefined genetic basis for breast development as it relates to extracellular matrix composition?
• **MEC substrate and ion transport**. Where are the major transporters for ions and nutrients localized in the lactating MEC?
• **MEC substrate and ion transport**. How is the function of the myriad substrate and ion transporters coordinated to produce milk of a consistent composition for the breastfeeding infant?
• **Endocrine regulation**. Why do we not see a consistent and dramatic improvement in milk production with PRL stimulating drugs like domperidone? https://www.ncbi.nlm.nih.gov/books/NBK501371/
• **Endocrine regulation**. How do hormone deficiencies or excesses alter milk volume and composition in breastfeeding parents?
• **Homeorhesis**. How do the key regulators of carbohydrate synthesis in the human breast—from environment and genetics, blood flow through to nutrient uptake, and enzyme concentrations—change during states of insufficient or sufficient milk production?
• **Homeorhesis**. How does blood flow to the breast regulate nutrient supply or uptake; does it co-modulate water uptake at the basal surface?
• **Microbiome**. Does the parental microbiome affect breastmilk production? To what extent does the infant microbiome reflect the parental microbiome in the exclusively breastfed infant?
• **Chronobiology**. What is the mechanism by which the central circadian clock impacts the mammary genomic clock and milk composition in humans? Is it affected by lactating parental factors such as artificial light, shift work, or obesity?
• **Chronobiology**. How do hormones that are important peripheral drivers of circadian biology—such as ghrelin, leptin, and PRL—change in human milk over the day?
• **Chronobiology**. Do circadian changes in milk have any functional significance for entraining infant circadian biology?

Alt-text: Text Box 4

## Lactating Parent-Infant Interaction As an Essential Process with Emphasis on the Contribution of the Lactating Parent

**FIGURE 2:**
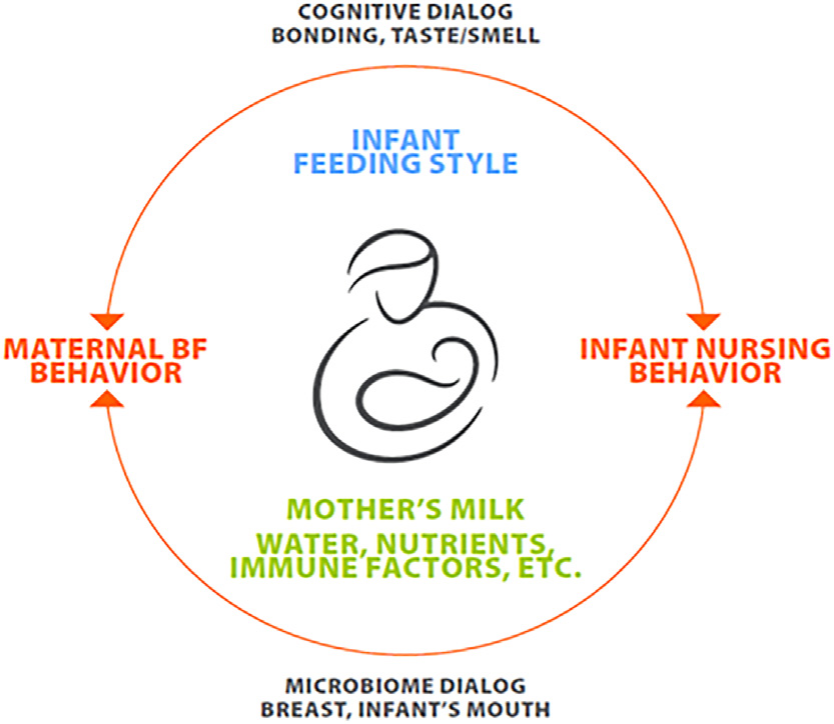
Lactating parent-infant interactions in the breastfeeding (BF) dyad.

Breastfeeding an infant creates an intimate dialog that leads to hormonal and other responses affecting numerous organ systems in the nursing dyad. This dialog includes both uni- and bidirectional components (Figure 2), the most obvious of which is the transfer of the lactating parent’s milk, with its complex array of nutritional, immunological, and other components, to the nursing infant. The infant’s feeding style in general (described in detail in the report of WG 3 [55]), as well as their mental and physical state during each nursing episode, are additional unidirectional components, as is the health status and mental state of the lactating parent. These elements create subsequent responses in both the lactating parent and infant that affect the brain in both individuals.

Descriptions of the normal biology of lactating parent-infant interactions are far from complete even for breastfeeding mothers, who have been studied most extensively. An important consideration is that a majority of lactating parents in the United States use a breast pump to collect their milk for feeding at a later time, both for personal reasons as well as to remain in the paid workforce [[95], [96], [97]]. The problem is that use of a pump modifies or interrupts the at-the-breast interactions depending on how breastmilk is fed to the infant (i.e., from a bottle) and who is delivering it [98]. Pumped milk also has a different composition from milk fed directly from the breast [99,100] (see also the report of WG 2 [64]). These facts lead to the questions stated in Text Box 5.

Text Box 5 **Lactating parent-infant interactions.**

• Does human milk collected by pumping affect the growth, health, and cognitive development of infants relative to breastmilk fed at the breast?
• Once milk is pumped, it is often donated, shared, or sold. Does consumption of shared or purchased milk affect the recipient infant’s health?
• How do the hunger factor ghrelin (present in breastmilk) and the motor factor of thirst, angiotensin II (present at high concentrations in neonatal plasma), interact to alter the cerebral oxytocin signaling system?
• Are the oxytocin receptor pathways in the human brain similar to those in rats, and how do they evolve with pregnancy and during lactation?
• How can communication between neuroscientists working on the pleasure-reward system and breastfeeding experts be enhanced?

Alt-text: Text Box 5

**FIGURE 3:**
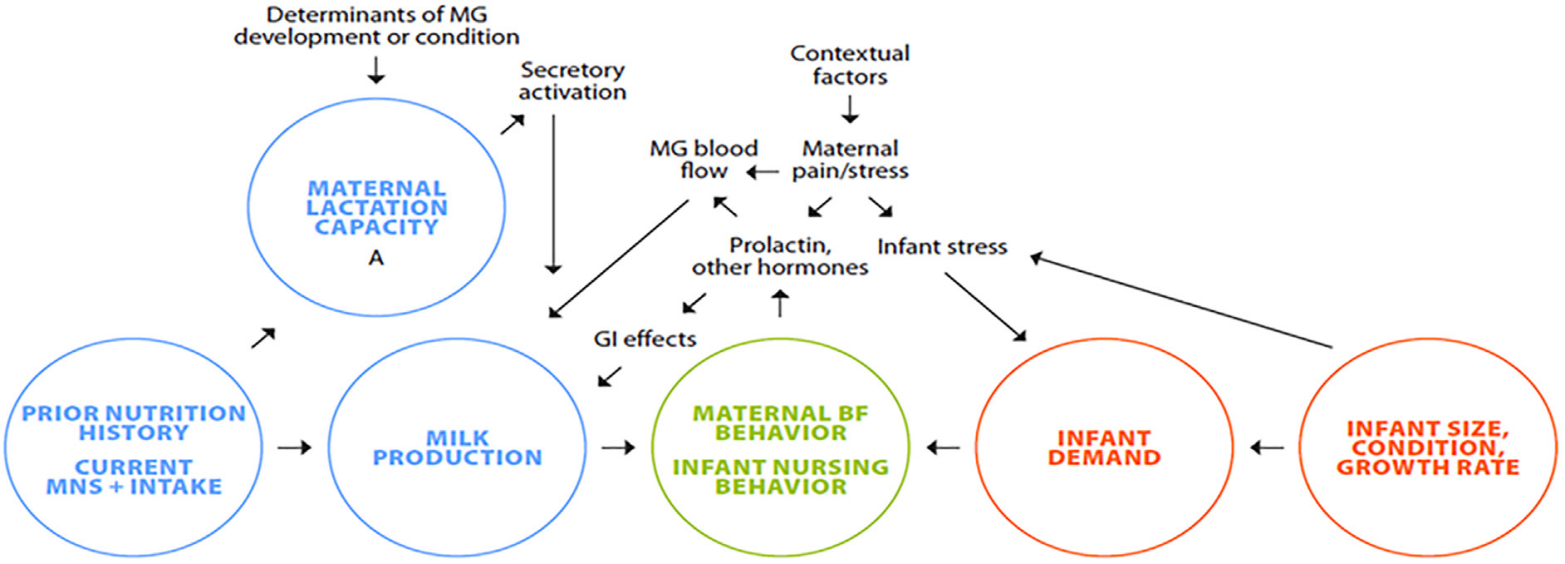
Factors that influence milk output. GI, gastrointestinal effects; MG, mammary gland; MNS, maternal nutritional status.

The lactating parent-infant dialog sits within a larger context (Figure 3) that is shaped, on the infant side, by their growth rate, attained size, and health, which are all experienced by the lactating parent as the infant’s demand for milk. Copious milk production is initiated in the first few days after birth during secretory activation. The ability of the gland to make milk, e.g., its “lactation capacity,” depends on the efficient utilization of nutrients to make sufficient milk volume to sustain infant growth and development. Potential factors that influence these variables include retained placental fragments that secrete progesterone, which inhibits secretory activation, altered secretion of placental factors that stimulate or impede breast development (see section on breast development) [101], and oxytocin secretion to produce milk letdown [102,103]. In women with obesity there is also inadequate mammary development, as well as impairment of both secretory activation and milk secretion [104].

Milk production is altered in response to feeding, which in turn meets infant demand in response to both the frequency and duration of sucking episodes. In addition, stimulation of the nipple areola complex sends messages to the brain and pituitary that regulate the pulsatile release of oxytocin and PRL [105]. The amount of the gastric hormone gastrin also increases rapidly in the mother with suckling; somatostatin is variable but shows an overall decrease by 60 min post-suckling [106]. The expulsion of milk by oxytocin-induced myoepithelial contraction moves milk from the alveoli, ducts, and sinuses. When this ejection fails to happen, the collecting ducts distend, and milk production is reduced [107]. In mice, if distention lasts beyond 2 days, apoptosis of secretory cells begins [108]. In humans the process is more difficult to study but in one careful histological study essentially no markers of lactation remained 3 mo after weaning [20].

## Oxytocin synthesis

**FIGURE 4:**
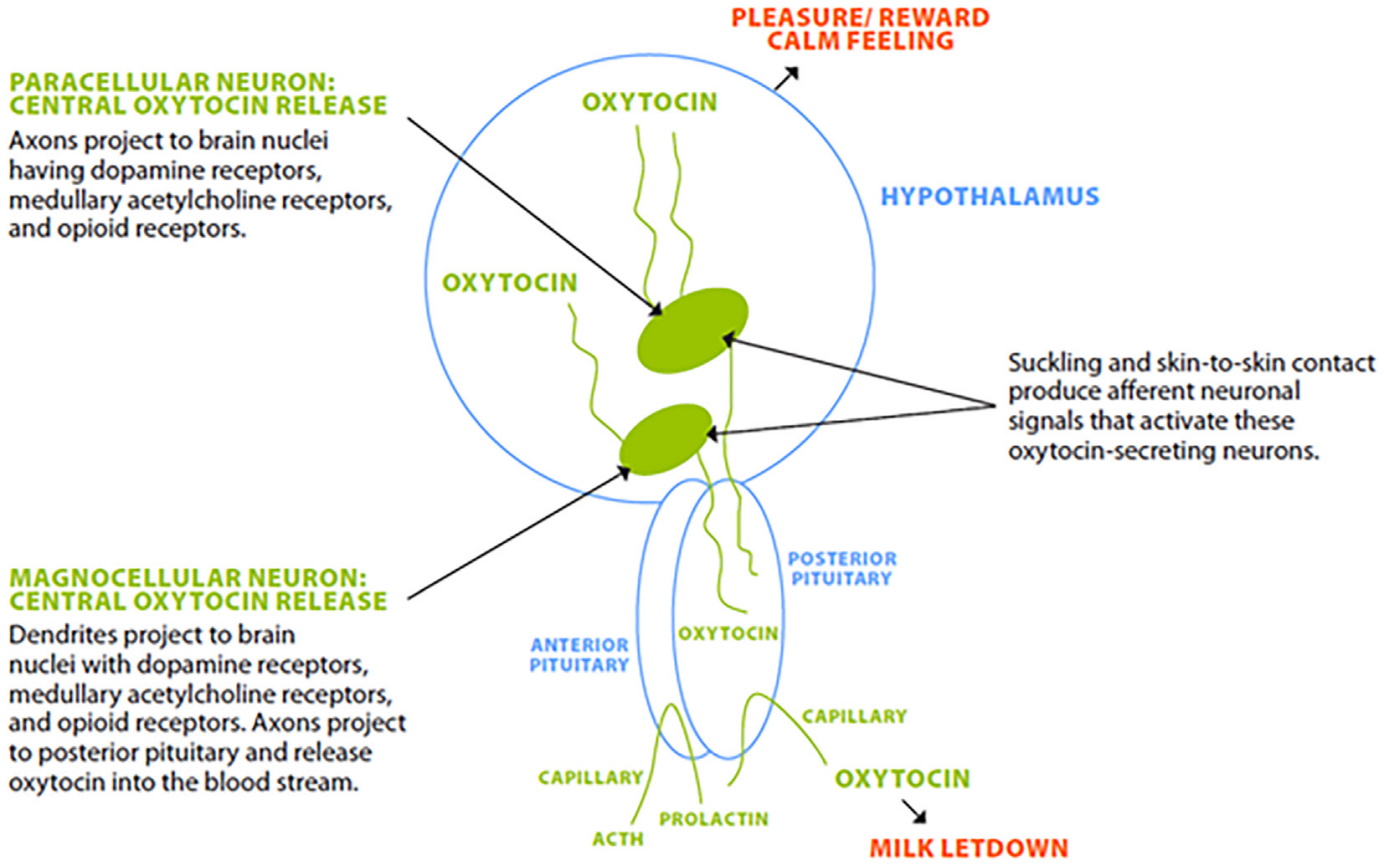
Hypothalamic oxytocin-secreting neurons simultaneously release oxytocin into the posterior pituitary, where it enters the bloodstream and the hypothalamus where it interacts with a specific set of neurons with oxytocin receptors, promoting lactating parental-infant bonding [103].

In addition to the letdown reflex, oxytocin also has complex interactions with neuronal centers in the hypothalamus [108]. Thus, oxytocin is synthesized in neurons in the paraventricular and supraoptic nucleus of the hypothalamus (Figure 4). Both suckling and skin-to-skin contact with the infant stimulate oxytocin production during breastfeeding, and the oxytocin is released *simultaneously* into the circulation and into the brain. One set of neuronal projections is directed to the posterior pituitary where the oxytocin enters the blood stream and, during lactation, produces the letdown reflex [109]. Another set of projections is directed to areas of the hypothalamus where the released oxytocin interacts with oxytocin receptors influencing postpartum behavior. These pathways have been studied extensively in rats, and good evidence indicates they also operate in the human brain, given that oxytocin receptors have been found on dopaminergic and muscarinic acetylcholine neurons as well as on neurons bearing opioid receptors in human brains [108]. Release of oxytocin into the brain promotes lactating parent-infant bonding, reduces anxiety and fear, and possibly decreases plasma cortisol [110]. One of the results of these interactions is that oxytocin is considered by some prominent psychologists to be “the initiating chemical messenger of the maternal infant tie” [111]. The importance of this network is highlighted by the demonstration that nursing mothers who abused drugs had a decreased response to oxytocin as monitored by magnetic resonance imaging [112].

From this overview, it is apparent that it is important to develop a more complete understanding of the factors that affect the capacity of the mammary gland to produce milk as well as the neural factors that lead to lactating parent-infant bonding. The results of this research could help more parents achieve their own and national breastfeeding goals and shed light on how to prevent problems in lactating parent-infant relations.

## Research Into the Effects of Common Clinical Conditions on Human Milk and Lactation Biology Requires Next Generation Methods

Approximately only about 25% of birthing parents in the United States breastfeed exclusively for the recommended 6 mo, and many do not meet their own breastfeeding goals [113]. Several health problems are implicated in these numbers, including inflammatory conditions, either localized as in mastitis, or generalized as in inflammatory diseases, all of which have significant negative impacts on milk composition and volume. Mastitis may increase the transmission of HIV-1 or other diseases to the infant or lead to bacterial dysbiosis in the infant gut [114]. Chronic inflammatory diseases such as Crohn’s disease impact milk composition in ways that appear to modulate milk components like secretory IgA [115]. Preterm birth, discussed extensively in the reports from WGs 3 and 5, may give rise to multiple effects on both milk composition and volume [116] and is often associated with defects in the metabolism of the lactating parent as outlined below. It is possible that changes in the secretion of extracellular vesicles by the placenta during gestation lead to alterations in mammary development such that milk composition may be compromised in those who give birth prematurely [117].

## Is human milk a source of infectious agents transferred from lactating parent to child?

In 2004, Robert Lawrence and Ruth Lawrence described the state-of-the-art data on infections and breastfeeding and concluded that “breastfeeding is contraindicated rarely during maternal infection [118]. The few exceptions are specific organisms with clear evidence of transmission through breast milk that cause significant morbidity and mortality because of infection through breast milk” [119]. Although infants are subject to infection with many types of bacteria, the only ones where evidence indicates transmission through breastmilk are *Mycobacterium tuberculosis* (MTC) and certain Streptococci. However, transmission of both could actually be through lesions on the breast. No MTC was found in the breastmilk of 18 women diagnosed with tuberculosis in Kenya [119]. Group B streptococcal infection is common in sub-Saharan Africa, and there is some evidence for the presence of the bacteria in breastmilk [120,121]; however, it is not clear that this is the major mode of transmission.

Five viruses that have clearly been shown to be transmitted in breastmilk include HIV type 1 (HIV-1), cytomegalovirus (CMV), and human T cell leukemia virus type 1 (HTLV-1), dengue virus (DENV), and Zika virus (ZIKV) [122]. The mechanism of secretion differs for most of these viruses: HIV-1 may be transcytosed across the mammary epithelium; HTLV is likely carried across the epithelium by leukocytes; and CMV is reactivated in mammary cells and shed into the milk. The probable mechanism of secretion of arboviruses such as DENV and ZIKV is not yet known, but these viruses must have some way of crossing the mammary epithelium into milk [123]. Arboviruses are transmitted by arthropod bites. Some like West Nile virus (WNV), ZIKV, DENV, yellow fever virus (YFV), and chikungunya virus (CHIKV) and many others can cause severe human disease, and ZIKV has been associated with severe outcomes in fetuses (called congenital Zika syndrome) [123]. Transmission of ZIKV in milk has recently been experimentally studied in mice where it is clear that this virus can be transferred from lactating parent to infant where it causes infection [123]. However, this study is the exception; viral transmission from breastfeeding parent to child has in most cases not been carefully studied.

Breastmilk components like lactoferrin or viral antibodies can neutralize viruses in milk or block their interaction with infant epithelia that come in contact with the milk. FFAs and monoglycerides in breastmilk inactivate enveloped viruses like SARS-CoV-2 and many others. This mechanism could account for the absence of SARS-CoV-2 in breastmilk [124]. Lactoferrin as well as many other compounds in breastmilk may interfere with virus binding to infant intestine or other cells, preventing infection in the infant. An understanding of how these mechanisms work may help in developing strategies for preventing viral infections by donor milk.

## Obesity and diabetes

The most prevalent clinical conditions known to alter human lactation and milk composition are obesity and diabetes [[125], [126], [127]]. Both are associated with significantly lower breastfeeding initiation, continuation, and exclusivity. Because breastfeeding by parents who are obese can be associated with obesity in the offspring [128], it is important to understand how obesity alters breast development and milk composition [129,130]. Certain metabolites as well as exosomal microRNAs have been proposed as agents that may mediate the associations between maternal and offspring obesity [[131], [132], [133]]. In addition, a growing number of epidemiologic studies report differences in the concentrations of leptin, insulin, adiponectin, and ghrelin [[134], [135], [136], [137], [138], [139]], pro-inflammatory cytokines [140], microRNAs, and HMOs [141] in breastmilk as well as changes in metabolomic profiles [134,142] in women with obesity and those affected by type 2 diabetes mellitus (T2DM) and gestational diabetes mellitus (GDM). However, little is known about how these diseases influence the ecological system of human milk [143].

The metabolic environment as well as the changes that accompany obesity and diabetes are complex, and many hormonal alterations likely contribute to impaired lactation, including systemic and localized elevations of adipokines like leptin, inflammatory cytokines, and insulin. Although MEC are thought to respond to insulin via the canonical insulin signaling pathway that includes the insulin receptor (IR), IRS1, and Akt [144], they do not depend on the insulin-stimulated glucose transporter, GLUT4, to take up glucose [145]. The IR is abundant in luminal progenitors isolated from human milk, but not in mature MEC [146], indicating a role for insulin in secretory differentiation as also seen in in vitro models of MEC development [147,148]. Mammary-specific manipulation of the insulin signaling pathway results in impaired lactation, whether signaling is inhibited [148] or overactivated [149]. These findings suggest that different pharmacologic therapeutics used to treat GDM (e.g., insulin compared with insulin sensitizers) will vary in their impact on mammary development [150].

Attention to other metabolic hormones during lactation has primarily focused on their presence in human milk and their potential effects on the offspring [137], whereas their effects on lactation remain largely unexplored. Serum leptin is positively associated with maternal adiposity as is increased local mammary adipose tissue leptin secretion [23]. Serum leptin is inversely associated with PRL concentrations in women [151]. In mice, diet-induced obesity in the pubertal period, but not in adulthood, resulted in elevated leptin expression in mammary adipose tissue along with stunted mammary ducts and reduced MEC proliferation [152]. Leptin receptor (LEPR) is expressed in the basal layer of the mammary epithelium in mice [153], but the LEPR transcript was not identified by scRNAseq in MECs isolated from human milk (personal communication, J. Martin-Carli & M. Rudolph), suggesting that leptin functions in the development and/or function of basally located mammary stem cells and myoepithelial cells. There is a critical need to understand whether the impaired lactation seen in human metabolic disease is mammary-specific or if its effects are primarily mediated via their actions on other metabolic organs.

We argue that current clinical studies provide suggestive evidence of alterations in mammary biology in parents with diabetes and obesity, and animal models provide mechanistic clues. However, as outlined in Text Box 6, we are still poorly equipped to precisely address how the systemic changes that accompany human disease act locally at the tissue or cellular level. In order to address these questions, next generation multi-omic approaches are urgently needed to advance the study of human milk and lactation as outlined extensively in the report of WG 4 [154].

Text Box 6 **Effects of common clinical conditions on human milk and lactation biology.**

• How do metabolic changes in obesity and diabetes affect mammary development?
• Does the context of pregnancy and lactation alter the metabolic effects associated with obesity and diabetes?
• Does obesity before or during lactation impact the “cross-talk” between MEC and white adipose tissue?
• Does the release of adipokines like leptin as well as inflammatory cytokines and chemokines like TNF-α and IL-6 alter mammary development or function?
• Does the hyperglycemia, hyperinsulinemia, hyperleptinemia, and systemic inflammation commonly observed in obesity and diabetes specifically alter cellular-level processes in the mammary gland, or are effects on lactation secondary?
• How is the cellular composition of human milk (e.g., secretory and immune cell profiles) regulated and how are they disrupted in common clinical conditions like mastitis or diabetes?
• What mechanisms control the enzymatic machinery that generates the diversity of lipids in human milk, and how is enzymatic function altered in different disease states?
• Do obesity and diabetes, before and during lactation (and their co-occurring conditions including depression and psychosocial stress), disrupt the oxytocin response to suckling?
• How can behavioral modifications, including management of gestational weight gain, improved diet quality, or stress reduction, reset lactation function to within the range observed in the normal weight, nondiabetic nursing adult?

Alt-text: Text Box 6

## Milk-derived mammary epithelial cells as a model for the study of milk secretion

The inability to directly access glandular tissue during lactation by biopsy is a substantial obstacle blocking mechanistic research into human lactation. A major advancement in the direct analysis of lactating MEC was achieved by defining the transcriptome of the MFG [29,30] that retains cytoplasmic fragments upon secretion to the lumen [155]. MEC in human milk are more amenable to experimental manipulations than MFG. However, the heterogeneity of the cell types present in milk complicates attempts to utilize these cells to their full potential, and it remains unclear how metabolically active these cells are.

This heterogeneity has recently been assessed comprehensively by single cell RNA-sequencing of milk-derived cells from exclusively breastfeeding women in several laboratories [146,156,157]. Immune cells, including T cells, natural killer cells, monocytes, macrophages, and dendritic cells have been identified. The remaining cells, a majority of those present, expressed luminal (KRT8 and KRT18) but not basal (KRT14) markers of the mammary epithelium. Most of the milk-derived MECs comprised fully mature secretory cells expressing high concentrations of transcripts encoding milk synthetic proteins, ribosomal machinery needed for high amount of protein synthesis, and many genes involved in de novo lipid synthesis. More recent transcriptomic analysis of milk cells has led to the identification of two types of luminal secretory cells and the analysis of changes in their genomes as lactation progressed [156, 157]. Although there has been great interest in purported stem cells in milk, the presence of stem cells expressing pluripotency factors OCT4, SOX2, and NANOG [158] has not been confirmed. Further, all nonimmune cells expressed the transcript for LALBA, indicating a lack of multipotent cells. In addition, the high degree of similarity between the transcriptomes of milk-derived MEC and the MFG suggests that MECs in milk represent many aspects of the luminal epithelium in situ and should be an important resource for analyzing the effects of many conditions of the birthing parent on the ecology of human milk.

Many questions remain regarding how various conditions might affect the types and functions of cells found in milk. Factors including time after delivery (colostrum, transitional, or mature milk) [[159], [160], [161], [162], [163], [164], [165], [166], [167]], term/preterm delivery [161,162,166,168,169], time relative to the feed (foremilk or hindmilk) [170,171], feeding frequency [172] as well as lactating parent/infant infections [159,160,162,163,[173], [174], [175], [176], [177]] alter the cellular concentration and/or composition of milk, although it is currently unclear how the cellular composition of human milk might be regulated or altered by obesity or diabetes.

## “Multiomic” understanding of lactation insufficiencies

**FIGURE 5:**
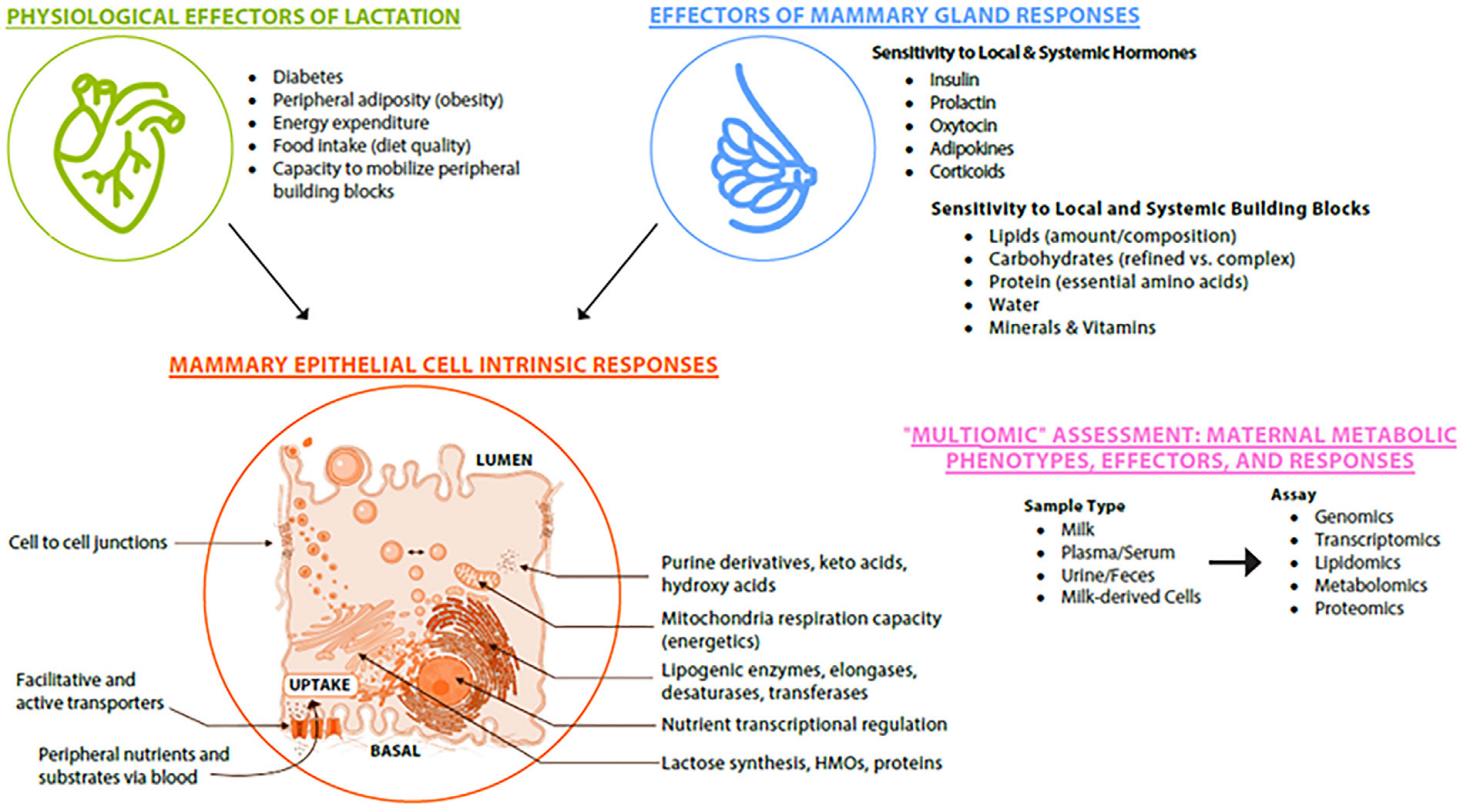
Metabolic status and adaptation for lactation: impacts on mammary epithelial cell (MEC) metabolism and identification using “multiomic” indicators of human milk, milk fat globules, and MECs. *HMO, human milk oligosaccharide.*

It is well established that parental changes in energy intake and energy expenditure, as well as mobilization of metabolic stores, are used to meet the biosynthetic demands of milk production. Lactating MEC have the prodigious task of taking up, synthesizing, repackaging, and eventually secreting vast quantities of all lipids broadly categorized as “milk fat” (Figure 5). For example, regulation of the enzymatic machinery generating the diversity of lipids in milk remains poorly understood, despite our ability to simultaneously detect >1600 validated lipid species [178,179]. This research gap is amplified when we consider the effects of the parent’s metabolic health on key endocrine regulators of the biosynthetic pathways controlling lipid biosynthesis such as PRL or insulin. Most clinical studies are designed to collect samples and data from lactating parents at the time of milk sampling, but then fall back on measures of parental pre-pregnancy BMI. To establish the causal chain connecting parental phenotype, MEC molecular function, and milk lipid/metabolite constituents, concordant analyses from both lactating parent and infant blood should be conducted alongside any analysis of MEC. Accordingly, the combination of unbiased measures (lipidomics, metabolomics, and transcriptomics) would provide simultaneous assessments of milk constituents and of the genes encoding enzymes needed for their biosynthesis. These analyses might then determine the relation to lactating parent circulating metabolic hormones as well as changes in MEC mitochondrial respiration.

## Connecting the dots

Recent technological advances have dramatically improved the ability of lactation biologists to understand the causal chain between metabolism in the birthing parent, MEC-intrinsic respiration, milk lipids and metabolites, and lipid/metabolites circulating in the infant’s blood. Several of these overriding questions are stated in Text Box 7.

Text Box 7 **Investigation of human milk cell properties: milk cell and multiomic analysis.**

• How do factors such as time after delivery, term/preterm delivery, time relative to the feed (foremilk, hindmilk, feeding frequency as well as lactating parent/infant infections) alter the properties of milk cells?
• Is the cellular composition of milk altered in lactating parents with obesity or diabetes?
• What are the key enzymatic pathways in human MEC that regulate milk lipidomic or metabolomic constituents?
• How are these pathways altered in MEC from milk of mothers with overweight, obesity, gestational and type 2 diabetes, as well as other common conditions?

Alt-text: Text Box 7

## Immune Profiling

### Immune tolerance in the human breast

Because the lactating mammary gland makes various proteins, carbohydrates, and lipids that have been absent in individuals who have not previously lactated or received human milk, they could incite an immune reaction if mechanisms for immune tolerance were not present in the lactating breast. These tolerance mechanisms are not well understood but may include the barrier function of the epithelium, which could be augmented by IL-17 produced by Th17 CD4+ T cells as found in some rodent models [179]. Schedin and colleagues emphasize that “The presence and requirement of mucosal immune programs in rodent models of lactation provide impetus to investigate the role of breast mucosal biology in supporting lactation in women” [180].

### Immune-related proteins in human milk

Immune factors in milk include both immune cells and soluble factors including immunoglobulins, growth factors, and cytokines. The most prevalent immune proteins in human milk are lactoferrin, lysozyme, secretory IgA, and soluble CD14, accompanied by a long list of proteins and cytokines whose function is unknown [181,182]. An unanswered question is how immune factors in milk relate to the development of food allergies in the breastfed infant [173].

Mastitis has been extensively studied in dairy animals [184], and lessons from that extensive literature may have relevance to human lactation. Certain immune factors associated with subclinical mastitis [185] have been extensively studied in women with HIV who mount a robust and prompt pro-inflammatory response to subclinical mastitis [186]. What is not clear is whether this response is characteristic of parents with other bacterial or viral diseases. A question of overriding importance in 2021 was whether SARS-CoV-2 or antibodies to this virus are transmitted in breastmilk. Definitive answers to both these questions have been rapidly obtained, and it is clear that transmission of this virus in breastmilk is rare. However, antibodies to the virus were present in the breastmilk of a majority of women who had had the disease, and in most cases these antibodies neutralized the virus [124]. These findings emphasize the rapid response of the milk immune system to a new pathogen in the environment of the lactating parent.

### Immune cells in the mammary gland and milk

Human milk contains activated neutrophils, macrophages, and T cells, although their role in the infant as well as the mechanisms by which they are secreted into milk are mostly unclear [187]. It has been shown that the adaptive immune system consisting of antigen presenting cells like CD11c cells and memory T cells such as CD4 Th1 cells, which secrete IFNϒ, is crucial for proper ductal development in the mammary gland of the pubertal mouse [188]. What we do not know is the role of CD4Th1 cells and IFNϒ in the developing human mammary gland.

### Immune cell imaging

Three-dimensional (3D) imaging of the murine mammary gland has revealed the interaction of immune cells with cells in the lactating mammary gland [189]. In these glands, cells stained with anti-CD45 [a pan-leucocyte marker] intimately associated with the myoepithelial cells, presumably providing the gland with enhanced immune surveillance. The number of CD45 cells associated with the epithelial layer increased ∼5-fold 0.5 mo after weaning as involution commenced [20]. Many questions remain as outlined in Text Box 8.

Text Box 8 **Immune proteins and cells in human milk.**

• Which types of leukocytes are associated with the various developmental stages of the human mammary gland?
• What is the functional role of leukocytes in mammary development and milk secretion?
• How do the types of immune cells associated with the mammary stroma, myoepithelial cells, and luminal cells relate to the types of immune cells in milk?
• How do immune factors vary with lactating parent factors such as diet, obesity, inflammatory disease (systemic or mastitis), probiotic ingestion by the lactating parent, ingestion or exposure to drugs and toxins, and the lactating parent’s genetic background?

Alt-text: Text Box 8

## Effects of Medications, Recreational Drugs, and Environmental Pollutants on Breast Development, Milk Secretion and Milk Composition

### Medications

Until recently the focus of lactation pharmacology has centered around questions such as “How much of the medication gets into breast milk?” “What percentage of the dose is absorbed by the infant?” “Does the absorbed dose of medication have physiologic or pathologic effects on the infant?” Although these questions remain relevant, there are important unanswered questions about the effects of medications, recreational drugs, and environmental pollutants on the quantity and quality of breastmilk. Four major issues in this regard are outlined in Text Box 9.

Text Box 9 **Effects of medications, recreational drugs, and environmental pollutants on breast development, milk secretion, and milk composition.**

• What is the actual extent to which lactating parents are exposed to prescribed and nonprescribed medications?
• Do medications alter the quantity and quality of the human milk delivered to the infant?
• Do medications, especially psychoactive medications, affect oxytocin/PRL release resulting from stimulation of the nipple by the breastfeeding infant?
• Do medications (i.e., psychoactive medications such as anti-depressants, antipsychotics, hypnotics, antihistamines, and narcotics) affect lactating parent-infant bonding?
• Do cannabinoids or disabling alcoholism change birth parent-infant bonding and the response to stress?
• Do cannabinoids or disabling alcoholism affect the letdown reflex?
• How do low doses of endocrine disrupting chemicals (EDCs) and their metabolites affect the pathways set in motion by estradiol, progesterone, hPL, PRL, and other agents during the development and function of the lactating breast?

Alt-text: Text Box 9

The most recent systematic review suggests that 60%–100% of women take at least one prescribed or over-the-counter medication in the first year after giving birth [190]. The surveys used often excluded the potentially important consumption of vitamins, nutritional supplements, and exposure to hormonal contraception. Further, these studies are limited by small sample size and, as documented in another study [191], failed to recognize that the parent was breastfeeding. Beyond the important issue of infant exposure and risk of adverse reactions to parental medications, there is a considerable lack of understanding concerning the effects of medication on lactational physiology, lactational neuroendocrinology, and the behavioral component of parent-infant bonding.

Sensory messages from the nipple and other sources in the breastfeeding parent reach the hypothalamus, which ultimately controls both oxytocin and PRL release (Figure 4). Pain, anger, confusion, and/or frustration are associated with a reduction in the normal pulsatile frequency of oxytocin and a 30%–50% reduction of milk volume per nursing episode. The major neurotransmitters are communicated to the brain by dopamine D_2_, serotonin 5HT_2a_, and/or serotonin 5HT_1A_ receptors. Many common antipsychotics such as the phenothiazines target the neurohormones associated with oxytocinergic pathways [192]. These drugs have been shown to disrupt attributes of maternal behavior including pup licking and pup retrieval in rodents [193]. The most popular antidepression medications utilize selective serotonin receptor uptake inhibitors (SSRIs) that delay secretory activation in women [194,195]; recommendations for their use in breastfeeding parents have not been formulated.

## Recreational drugs

Millions of Americans used or misused various products in 2018–2019 including tobacco (58.1 million), marijuana (48.2 million), psychotherapeutic drugs (16.3 million), alcohol (14.5 million), pain relievers (9.7 million), hallucinogens (6.0 million), cocaine (5.5 million), inhalants (2.1 million), methamphetamines (2.0 million), and heroin (0.75 million) [185]. In addition, between 10% and 60% of these individuals used one or more recreational drugs at the same time [196]. Lactating parents probably reduce their intake of recreational drugs and may change their pattern of feeding and/or may supplement with formula during periods of high drug use. The need for research in this area is illustrated by the finding that tetrahydrocannabinol and cannabidiol accumulate to significant concentrations in breastmilk relative to those in plasma [197]. The little that is known about their effects on lactation and the recipient infant are summarized by WG 3 [55], and research approaches are summarized by WG 5 [198]. Alcohol, particularly in disabling alcoholism, has mostly been studied for its transfer into breastmilk and its effects on the infant; this topic is well covered by WG 3 [55]. However, there are questions regarding its addictive use in the lactating parent because it is a neurotoxin that significantly changes parental behavior as well as depressing myometrial/myoepithelial function. Important questions about cannabinoids or disabling alcohol use in lactation are given in Text Box 9.

## EDCs

At least 2000 environmental pollutants have been identified. About 60% are EDCs including parabens, phenols, phthalates, metals, and polycyclic aromatic compounds. These EDCs can present as a primary chemical, a congener, or an active metabolite. EDCs penetrate most tissues and fluids in the body, i.e., blood, urine, placenta, breastmilk, and infant blood and urine, often at doses that can be worrisome because most EDCs have low-dose effects and nonmonotonic dose responses [15,199]. Exposure to EDCs is associated with dysfunction of hormone-mediated processes such as metabolism, energy balance, thyroid and reproductive functions, immune functions, and neurodevelopment. However, the focus of most case studies and case series has been the concentration of the chemicals in breastmilk. One excellent annotated compilation reviewed studies from 13 states in India [200]. Although that report contains detailed information on 101 environmental contaminants detected in human milk samples, as compiled from 36 research articles, the effects on milk production are largely undocumented. In 2015, The Endocrine Society published a review of the adverse effects of EDCs in men and women [201]. Conspicuously, the adverse effects of EDCs on lactation, breastfeeding, and milk production were absent.

In summary, we are at the early stages in understanding the systems biology of human milk and lactation. The parental and environmental inputs that affect lactation and milk composition intersect with questions of whether and how they are modified by depression, anxiety, and psychosocial stress. Experiences of racial discrimination and economic instability and their activation of physiological stress responses are at epidemic proportions in reproductive-age persons in the United States, but they remain severely understudied in relation to lactation. To this end, diversification of our study populations to include families of color and lower income families is critical.

## Funding

The BEGIN Project was initiated by the Pediatric Growth and Nutrition Branch of the *Eunice Kennedy Shriver* NICHD of the United States NIH in partnership with the Bill & Melinda Gates Foundation and the Academy of Nutrition and Dietetics (Academy). This supplement was supported by the *Eunice Kennedy Shriver* NICHD at the NIH. Support for assistance (by BioCentric, Inc.) with editing, proofing, and submitting the manuscripts was also provided by the *Eunice Kenedy Shriver* NICHD.
